# Estimation of *Ixodes ricinus* (Acari: Ixodidae) Populations of Kaylaka Park in the Town of Pleven, Bulgaria

**DOI:** 10.3390/insects12090808

**Published:** 2021-09-09

**Authors:** Alexander Blazhev, Milena Atanasova, Krasimir Kostov, Tsetsa Doychinova, Svetla Blazheva, Milena Karcheva

**Affiliations:** 1 Department of Biology, Medical University-Pleven, 1 Kliment Ohridski Str., 5800 Pleven, Bulgaria; milena.atanasova-radeva@mu-pleven.bg; 2 Department of Pathophysiology, Medical University-Pleven, 1 Kliment Ohridski Str., 5800 Pleven, Bulgaria; dr.krasi_kostov@abv.bg; 3 Department of Infectious Diseases and Epidemiology, Medical University-Pleven, 1 Kliment Ohridski Str., 5800 Pleven, Bulgaria; doichinova_ceca@abv.bg (T.D.); milena_karcheva@abv.bg (M.K.); 4 Department of Immunology, University Hospital, 5800 Pleven, Bulgaria; svetlabl@abv.bg

**Keywords:** *Ixodes ricinus*, tick collection, tick density, flagging, medical entomology, Kaylaka Park

## Abstract

**Simple Summary:**

Hard ticks transmit the etiological agents of numerous diseases. Kaylaka Park is a protected area, but part of it is designated for various outdoor activities. The aim of our study was to establish the presence of hard ticks in four urbanized areas and four areas that are not maintained and are natural wilderness areas (wild areas). The flagging method of collection was used. Temperature, relative humidity, both collection time and distance covered were measured during the sampling campaigns. The density of ticks collected was calculated, the number of ticks captured per minute was calculated and the results were compared between urban and wild areas over a five-year period (2016–2020). A total of 622 ticks were collected. All of them were identified as *Ixodes ricinus*. Significant differences between the urban and wild areas were observed in the number of ticks per minute and density of nymphs. The peak in questing tick activity has been established at the end of April. The highest yield was obtained at 20 °C and at 60% relative humidity. We found that the distribution of *Ixodes ricinus* ticks is widespread in Kaylaka Park. Their high density poses a serious risk to park visitors in both wild and maintained urban areas.

**Abstract:**

(1) Background: Ticks are vectors of a large number of pathogenic microorganisms, which cause serious diseases in both humans and animals. Kaylaka Park is located in northern Bulgaria close to the city of Pleven. Part of the park is urbanized and visited daily by many citizens. The aim of our study was to determine the presence and distribution of hard ticks in the park area by surveying and comparing four urbanized with four wild areas. (2) Methods: Ticks were collected by flagging from 2016 to 2020 during the spring–summer season (March–July). Air temperature, relative humidity, collection time and flagging area were measured during the campaign. (3) Results: A total of 622 ticks were collected: 285 females (46%), 272 (44%) males and 64 (10%) nymphs. All were identified as *Ixodes ricinus*. Wild areas showed statistically significant higher values of ticks collected per minute (*p* = 0.009) and nymph densities (*p* = 0.003) compared to urbanized sampling sites. Other densities indices did not have a significant difference between urban and wild areas. Highest numbers of *Ixodes* ticks were collected at a temperature of 20 °C and at 60% relative humidity. The active questing began in March, peaked in end of April and declined in June. (4) Conclusions: In the present study, we found that ecological factors in the Kaylaka Park area are favourable for the development and distribution of tick populations. The results give us reason to consider that there is a high risk to visitors from tick bites in the Kaylaka Park area.

## 1. Introduction

Hard tick species (Acari: Ixodidae) act as vectors of pathogens to humans and animals for several bacterial, viral and protozoan pathogens, causing public health risks [1,2,3]. Exophilic tick species, such as the most extensively studied and medically important species in Europe, *Ixodes ricinus* (the castor bean or sheep tick), quest on vegetation for meals on mammalian, avian, or other hosts in each developmental stage: larva, nymph and adult female (the adult male of some species may also feed on blood but does not fully engorge) [4]. When not actively questing, these ticks spend long periods off-host in the soil or leaf litter layer [5,6]. Studies have indicated that *I. ricinus* populations have increased significantly in many places over the last few decades, and they are expected to continue to grow [3,7,8].

In the last few decades, an increase in both tick populations and the number of observed cases of tick-borne infections have been reported in many European countries [9,10,11,12,13]. Climate change is thought to increase the spread of ticks in latitude and increase their abundance [14,15,16]. In fact, observations determined in recent years confirm these predictions [8,11,17,18,19]. In Bulgaria, over the last decade, data from the National Centre for Infectious and Parasitic Diseases have reported an increasing number of cases of tick-borne infections [20].

In European cities, public parks, gardens, cemeteries and peri-urban leisure-time areas have become particularly important places where humans and domesticated animals can encounter potentially infected questing ticks [3,21]. Publications related to tick populations in urban parks and gardens [3,22,23,24,25,26,27,28,29] show the presence of viable tick populations as well as the presence of tick-borne diseases in these areas. Research on tick populations in Bulgaria is scarce.

Kaylaka Park is a recreational park of 1000 hectares, which is considered a ‘green lung’ for the city of Pleven. It is located in the karst valley of the river Touchenitsa. Kaylaka Park is a local hotspot for leisure activities with a large number of daily visitors, especially during the warm seasons of the year. This type of resting area is of growing scientific interest in tick density and tick-borne pathogens [27,30]. The park is located on the periphery of the city. At a distance of about 2.5 km from the entrance of the park, there are many attractions for citizens, such as cafes, a zoo, a theatre, sports fields and playgrounds. This part is visited daily by people with various outdoor activities. The lawns are mowed and maintained regularly. The rest of the park belongs to the protected area “Kaylaka”, where maintenance is only carried out around the large infrastructure sites. The flora and fauna are relatively natural with minimal human intervention.

In our previous retrospective study on tick-borne disease [31,32], we found that there is an increase in registered cases of Lyme borreliosis in the region of Pleven. Additionally, many cases of tick bites have been reported in the Kaylaka Park and protected area. These data led us to the idea of studying tick populations in this area, as a possible foci and a potential source of infections.

Poor data regarding the hard tick fauna and tick-maintenance wildlife hosts in urban areas in Bulgaria have motivated this research. The study aims to contribute to a better knowledge of the ecological factors which impact the diversity and abundance of hard ticks; consequently acting as major hazard determinants of tick-borne diseases in urban environments.

The objective of the present study is to provide a preliminary image of the abundance of the questing ticks in urbanized and wild areas in Kaylaka Park—Pleven (Bulgaria), using the flagging method of sampling.

## 2. Materials and Methods

### 2.1. Study Sites

Eight sampling sites in Kaylaka Park were investigated over a period of five years (2016–2020). The sites in Kaylaka were divided into two categories, urban and wild area, according to the daily presence of people and the transformation of the area. The urban area included four sites located closer to the city of Pleven (U1–U4) and the wild area included four sites in the park more distantly situated (W1–W4). The first group is with a daily visit of citizens and high human-derived landscape transformation, involving highly transformed areas of urban infrastructure, residential areas, streets or arable land, including playgrounds, cafes, restaurants, alleys for sports and more. The second category includes semi-natural woodland areas where natural and managed forests and low-transformed settlement foci, with lesser population visit.

#### 2.1.1. Urban Area

Sampling sites U1 to U4 (central alley 2.8 km) are an alley within the entrance of the park. On both sides of the alley, there are various deciduous and coniferous trees and meadows. Urban sites are under the strong anthropogenic influence, highly frequented by visitors and dogs, maintained by gardening (e.g., mowing) and intensively used by visitors for different outdoor activities especially regularly walking dogs, walking with children, cycling, etc. (Table 1).

All urban sites are inhabited by rodent and avian hosts, as well as synanthropic carnivores such as dogs (*Canis lupus familiaris*), European polecat (*Mustela putorius*) and hedgehogs (*Erinaceus europaeus*). Larger mammals such as roe deer (*Capreolus capreolus*) may also occur (personal observation during study period) [33].

#### 2.1.2. Wild Area

Wild sites are known for being inhabited by numerous large mammals, particularly a free-living population of the roe deer (*Capreolus capreolus*) or wild boar (*Sus scrofa*). Jackals (*Canis aureus*), foxes (*Vulpes vulpes*), European badger (*Meles meles*) are permanent inhabitants of the forest, which is also the habitat of many rodents, insectivores, a large number of bird species and reptiles (personal observation) (Table 1) [33]. The wild sampling sites have a much lower level of everyday activity of humans and are visited mainly by forestry workers, tourists, collectors of herbs or mushroom pickers, during selected periods of the year.

### 2.2. Tick Collection and Identification

Tick sampling campaigns were performed from March to June, in a 5-year period, between 2016 and 2020 using the flagging method with a flannel flag (dimensions around 100 × 100 cm) mounted on a handle. The exact distance flagged at each sampling site during any single visitation depended on available time and weather conditions, as well as local tick density (generally less dragged in high density areas). Flagging spots in sampling sites were selected separately for each session, based on the operator’s assessment of suitable tick microhabitats, and covered varied areas and biotopes within the sampling sites. This method only collects ticks that are questing in the upper parts of the vegetation. Ticks caught in this manner represent only a small fraction of the total population, as ticks periodically return to lower vegetation levels to regulate their water balance [34,35]. The days of sampling were chosen using the following criteria: rainless, wind speed less than 3 according to Beaufort wind force scale, air temperature above 12 °C. For this, a weather station (Auriol IAN 94,604 OWIM GmbH & Co. KG, Neckarsulm, Germany) was placed on the ground in each flagging area to monitor local temperature and hygrometry. Total sampling time and surface area were recorded for all sampling events. The time spent on flagging ranged from 5 to 60 min, including time needed to extract ticks from the flag with tweezers to put them separately in 1.5 mL safe-lock Eppendorf tubes (Eppendorf AG, Hamburg, Germany). Collected ticks were transferred to the laboratory where they were washed with sterile water in ultrasonic cleaner (SilverCrest, HOYER Handel GmbH, Hamburg, Germany), identified to the species level, developmental stage and gender according to the morphological keys provided in Georgieva [36], Filipova [37] and Estrada-Peña et al. [38] using a stereo microscope (Olympus SZ4045, Olympus American Inc., Melville, NY, USA).

### 2.3. Questing Tick Abundance

Tick density was expressed as the number of ticks caught per 1 m^2^ drag. Ticks were counted and their densities were calculated per 1 m^2^ for each individual flagging event (each visit at specific site). The flagging area was calculated using the “Measure distance” tool of the Google Maps application after each collection campaign.

Since flagging in different sampling sites was performed unevenly (different time, duration of flagging and a different number of samplings), to estimate and compare questing tick abundance in the different study areas, we introduced several indices that reflect the density of questing ticks. The collected questing ticks were converted into a questing index (QI), as the number of collected ticks from the vegetation was divided by the flagged area. QI was calculated for *I. ricinus* ticks (separately for adult, nymph, location and flagging event), as follows: the adults (AQI) (males and females were considered together), the nymphs (NQI) and the total number of *I. ricinus* ticks per location (TQI). The conversion was performed according to the formula: QI = *n*/S, where *n* represents the number of collected ticks and S is the surface of the sampled area in square meters. The area and corresponding indices were calculated immediately after each sampling. The resulting indices were used for statistical analysis. The average area of each sampling site and the corresponding indices over the five-year study period is shown in Table 2.

Another abundance parameter that was calculated for each location individually is the ticks per minute (TM). The TM represents the total number of ticks collected in each location regardless of stage and sex for a minute. The total number of ticks collected was divided by the time of flagging in each event.

### 2.4. Statistical Analyses

Statistical analyses were performed using SPSS 23.0 (SPSS, Inc., Chicago, IL, USA), Prism version 8.0.0, (GraphPad Software, San Diego, CA, USA) and Microsoft Excel (2007) for Windows. To test whether values follow a Gaussian distribution, the Shapiro–Wilk normality test was used. The data were expressed as mean ± standard deviation (SD). The differences between the urban and wild sampling sites were assessed by Mann–Whitney U-test (nonparametric data distribution). Analysis of variance (ANOVA) was conducted to compare mean tick indices TQI, AQI, NQI and TM with RH and temperature of the environment (parametric data distribution). Pairwise comparisons were carried out using the Tukey post hoc analyses. Values of *p* < 0.05 were considered statistically significant.

## 3. Results

### 3.1. Tick Abundance

In the Kaylaka Park, surveillance campaigns were performed monthly, a few days apart, in March, April, May and June, for a period of five years (2016–2020), when meteorological conditions fulfilled the criteria of the study design. No sampling was performed after July due to the particularly hot and dry weather conditions. A total of 622 ticks were collected. One hundred and ninety-seven were collected from the urban area (52 in U1, 66 in U2, 37 in U3 and 42 in U4), 425 were collected from the wild area (149 in W1, 94 in W2, 49 in W3 and 133 in W4). All of the ticks were identified as *Ixodes ricinus* (of which 46% (*n* = 285) were female, 44% male (*n* = 272) and 10% (*n* = 64) were nymphs). The data were obtained from a total of 42 tick collection campaigns performed 15 times in the wild and 27 times in urban areas. The overall mean indices calculated over five years of study for *I. ricinus* distribution is presented in Table 2. 

**Table 2:** The number of *Ixodes ricinus* ticks collected and the indices for total, adults, nymphs and ticks per minute in the urbanized and wild areas. The data presented are average values ± standard deviations (SD) for a five-year study period (2016–2020).

Sampling Sites	Collected Ticks				Average Flagging Size (m^2^)	Average Flagging Time (minute)	*I. ricinus* Abundance Indices (Mean ± SD)			
	Total	Female	Male	Nymph			TQI	AQI	NQI	TM
**Urbanized**	197	101	83	12	302.26 ± 706.72	17.35 ± 14.14	0.054 ± 0.089	0.051 ± 0.089	0.003 ± 0.005	0.579 ± 0.669
**U1**	52	27	20	5	344.62 ± 299.97	23.4 ± 11.4	0.034 ± 0.028	0.03 ± 0.025	0.004 ± 0.007	0.335 ± 0.212
**U2**	66	36	25	5	223.90 ± 106.91	14.6 ± 3.2	0.041± 0.030	0.037 ± 0.026	0.004 ± 0.006	0.491 ± 0.271
**U3**	37	15	20	2	322.27 ± 176.39	17.2 ± 17.3	0.057 ± 0.080	0.056 ± 0.081	0.001 ± 0.002	0.577 ± 0.201
**U4**	42	23	18	0	364.17 ± 289.17	14.0 ± 10.0	0.102 ± 0.189	0.102 ± 0.189	ND	1.08 ± 1.452
**Wild**	425	184	189	52	773.97 ± 736.82	27.5 ± 13.71	0.087 ± 0.097	0.073 ± 0.076	0.017 ± 0.026	0.971 ± 0.538
**W1**	149	65	66	18	450 ± 0	30.7 ± 14.0	0.11 ± 0.068	0.097 ± 0.06	0.013 ± 0.008	1.516 ± 0.396
**W2**	94	39	37	18	196.67 ± 148.36	20.3 ± 6.3	0.136 ± 0.128	0.113 ± 0.093	0.031 ± 0.038	0.865 ± 0.427
**W3**	49	22	22	5	644.72 ± 354.63	20.3 ± 12.4	0.016 ± 0.017	0.014 ± 0.016	0.002 ± 0.002	0.608 ± 0.735
**W4**	133	58	64	11	2424.87 ± 1978.76	48.3 ± 23.6	0.06 ± 0.08	0.048 ± 0.06	0.012 ± 0.019	0.96 ± 0.131
**Total**	622	285	272	64	481.98 ± 736.82	21.21 ± 13.71	0.066 ± 0.092	0.059 ± 0.084	0.008 ± 0.018	0.717 ± 0.641

U1–U4: urban areas 1–4; W1–W4: wild areas 1–4; TQI: total questing ticks index; AQI: adult questing ticks index; NQI: nymph questing ticks index; TM: ticks per minute; ND: no data.

**Figure 1:**
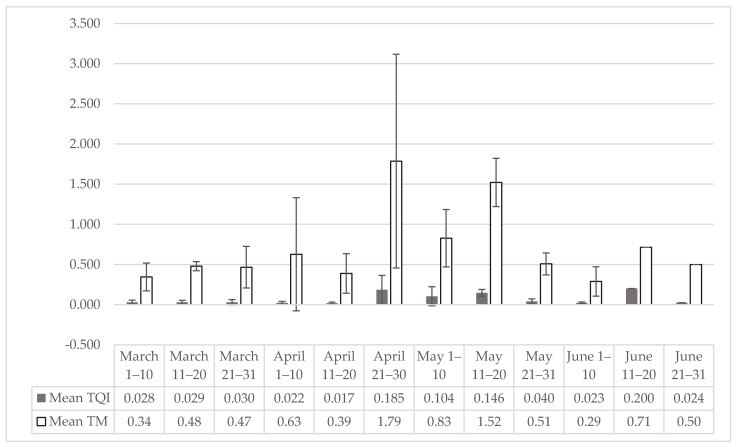
Dynamic of questing activity of *I. ricinus* represented by mean ± SD (TM and TQI) over the 5 years by 10 days period from March to June. TQI: total questing ticks index; TM: ticks per minute. Error bars represent standard deviations.

The active questing activity of *I. ricinus* began in March, peaked in end of April and declined in June. Using the mean values of TQI and TM for a period of five years, we detected the highest activity within the last 10 sampling days in April (Figure 1 and Table 3). No autumn activity was surveyed due to unfavourable weather conditions and impassibility at some of the sampling sites.

### 3.2. Temperature and Relative Humidity (RH)

**Figure 2:**
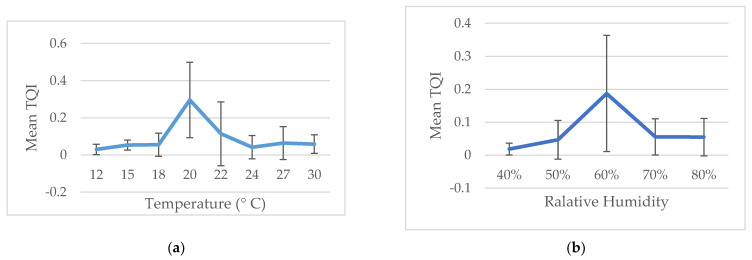
TQI ± SD over environmental conditions during the tick’s collection campaign (2016–2020). (**a**) The influence of temperature on the TQI; (**b**) the influence of RH on the TQI. Error bars represent standard deviations.

No statistically significant differences were found when comparing TQI, AQI, NQI and TM with temperature and RH (one-way ANOVA). Our result showed that tick questing activity was observed between 12 °C and 30 °C with a peak of maximum activity at 20 °C. The RH at which the collections were performed varied between 40% and 80%, with the detected peak of questing at 60% RH. Due to uneven tick collections from different sampling sites and months, temperature and RH data were grouped into ranges and plotted against the TQI (Figure 2).

### 3.3. Comparison of Questing Indices among the Urban and Wild Areas in Kaylaka Park

Wild areas showed statistically significant higher values of TM (*U* = 107, *p* = 0.009) and NQI (*U* = 101.5, *p* = 0.003) compared to urbanized sampling sites. Other indices did not show significant differences between urbanized and wild sampling sites (TQI: *U* = 157, *p* = 0.187; AQI: *U* = 158, *p* = 0.195). The detailed results obtained from the comparison between all urban and wild areas are presented in Table 4.

## 4. Discussion

The purpose of this study was to evaluate the populations of hard ticks (Ixodidae) in urbanized and rural areas in Kaylaka Park in the city of Pleven (Bulgaria). Until now, no studies of tick fauna have been conducted in the area of Pleven region and Kaylaka Park.

The increased risk of tick bites in urban parks and gardens has been studied in many European countries [3,8,39,40,41]. In Bulgaria, the first studies of tick populations in an urban environment were conducted in Sofia [42]. Publications related to the presence and distribution of ticks in other parts of Bulgaria are scarce.

A number of studies show that ticks now find favourable conditions in urban green areas, in addition to pastures, meadows and forests. Stable tick populations have been reported from different city parks in Europe and many pathogens responsible for causing disease in humans and domestic animals have been detected in ticks collected from those peri-urban areas [24,26,27,39,41]. Parks and gardens during the spring–summer season are visited and entertained by many citizens each year; it is, therefore, necessary to fully assess the risk of tick bites in order to prevent this major threat to public health.

We confirmed the presence of ticks in all surveyed areas in Kaylaka Park, with no significant differences in TQI between urban and wild sampling sites. *Ixodes ricinus* dominates the tick fauna in Europe and is also the most-studied species [43]. Its role as a vector has been proven for various pathogens such as *Borrelia* spp., *Anaplasma phagocytophilum*, *Babesia* spp, tick-borne encephalitis virus, etc. [8,27,29,40,42].

Lack of differences in *I. ricinus* TQI between urban and wild areas in the protected area of Kaylaka are most likely due to the large number of small and medium-sized mammals and the many birds inhabiting the park area. Most of the ticks collected in urban areas have been found near places where there is food waste, near bars, restaurants, playgrounds and trash cans. These places attract small rodents as well as stray dogs which are the hosts in the different stages of the ticks [30]. On the other hand, the variable sampling regime, uneven distribution across months and sampling sites could lead to a lack of differences in TQI between urban and wild areas.

The occurrence and abundance of a certain tick species in a given habitat are mainly determined by abiotic factors, habitat characteristics and availability of hosts [44]. *Ixodes ricinus* is an exophilic three-host tick species restricted to areas of moderate to high rainfall with vegetation that retains high humidity. During off-host periods, *I. ricinus* requires a relative humidity of at least 81–85% to survive [45]. Kaylaka Park is located in the canyon of the small river Touchenitsa, and on both sides is surrounded by rock formations, which provide a relatively constant humidity in the park. On the other hand, the park is bordered by wooded areas inhabited by various animals, delivering an excellent condition for the life cycle of the *I. ricinus* ticks in all stage of its development.

An interesting fact is that 90% of the collected ticks were adults. The few nymphs caught and the lack of larvae could be explained by the flagging method. Nymphs, due to their significantly smaller size are more sensitive to water loss and possess lower absolute energy reserves than adults [46]. Due to this, few nymphs climb to the top of the vegetation, which reduces the chance of being captured by flagging. It is likely that many of the nymphs are in the lower layers of vegetation that remain untouched by the flag. Nymphal activity may be greater in the autumn, when no collecting campaigns were conducted, due to unfavourable weather conditions and inaccessibility at most wild sampling sites in the Park. The highest questing activity was observed in the period from the end of April to the beginning of June. This activity was represented both by the TQI and by the collected ticks per minute (Figure 2a,b). Indirectly, we assumed that the main activity of ticks was the spring period. In our previous retrospective study of tick-borne infections in Pleven, it was found that the registered cases of Lyme borreliosis and tick bites were mainly in March–July and only isolated cases in the autumn season [32]. The climate in Northern Bulgaria is characterized by hot, dry and long summers until mid-October, after which temperatures drop to critical for tick questing activity [47]. Perhaps there is some minimal autumn tick activity, but the weather did not allow appropriate conditions for flagging and ticks collecting due to rain, heavy wind and impassability in some of the areas at this time of year.

The biggest challenge for terrestrial arthropods is to maintain water balance, a fact that is particularly important for the survival of ticks and especially for the *I. ricinus* as it is extremely sensitive to temperature and relative humidity compared to other hard tick species [48,49]. Similar observations were determined in the present study. The increase in relative humidity had a positive effect on the activity in questing *I. ricinus*, as the maximum activity expressed by TQI reached up to 60%. The decrease in TQI at an elevated relative humidity seems contradictory at first glance, possibly due to the longer search activity and greater chance of attachment to hosts. Because of the larger number of ticks that attach to a host, the number of questing ticks on the grass surface is depleted and results in their lower amount at flagging. Compared to the temperature, the TQI increases and reaches its highest values at 20 °C, after which the TQI falls (Figure 2a). The rise in temperature has a negative effect on the survival of ticks, as well as their activity [50]. Therefore, after June, we did not find ticks in the park area. The higher questing activity in spring in Kaylaka Park was probably due to the complex effect of local climatic factors and the duration of the day. During the five-year study, we did not survey fall activity.

Ticks per minute (TM) was an index we used to estimate the chance for a questing tick to attach to a host (human or animal) in case a likely host spends a specified time in a given area. We found this index more relevant than the tick density because collection times could vary across areas of equal dimensions. This index indicated the frequency of ticks in an area, i.e., the number of ticks collected per time unit. On the other hand, TM provided information about the rate at which questing ticks were collected by flagging from a certain sampling site.

In the present study, we found significantly higher values of TM in the wild areas compared to the urban ones. Which means that in urban areas, the frequency of ticks was lower than in wild areas. A short stay in wild area was required until a tick was caught. When comparing the zones between them, significant differences were found between U1 (0.335 ± 0.212) and three of the wild zones W1 (1.516 ± 0.396), W2 (0.865 ± 0.427) and W4 (0.96 ± 0.131). There were no significant differences between urbanized areas, except when comparing TM between U1 and U3 (0.577 ± 0.201). The higher TM value at the U3 sampling site could be explained by the vicinity of the U3 to the zoo. (Table 4). The large mammals in the zoo are also a source of blood meal for the ticks. Despite the insect and tick control measures, this probably influences the higher number of ticks in the area around the zoo.

Falco and Fish [51] studied recreational areas for the risk of acquiring a tick bite and possibly Lyme disease in parks in New York. They use an index called “encounter distance”. It measures the distance that the first tick will catch on the flag. Their results also show that in areas of parks that are used more often by citizens, there is a lower risk for tick bites, as more walking distance is required to catch a tick.

It is difficult to compare the two indices, but in both studies the aim was to determine the risk to humans. With an encounter distance, it is possible to catch a tick on the flag in the first step, but the next questing tick will be after many meters away. For this reason, we chose the risk to be assessed by the capture number per flagging time. On the other hand, the time spent staying or walking in a habitat inhabited by ticks significantly increased the risk. Although there were no differences in densities between urban and wild areas, comparing the number of ticks per unit time shows that in wild areas there was a higher risk of tick bites.

Finding fewer ticks in frequently used urban areas could result from ticks attaching to domestic dogs and humans and being removed from the questing population [52]. Additionally, this may be due to the more frequent maintenance of green areas such as mowing and exporting green waste. On the other hand, fewer ticks in high-use areas may be due to habitat alteration, such as buildings, roads and maintained lawns, which may discourage wild mammal hosts or reduce tick survival.

The tick questing index in urban and wild areas did not show statistically significant differences, indicating that the risk of tick bites was almost the same for both types of areas. However, a significant difference between wild and urban areas was shown by NQI, as well as the number of ticks caught per minute. Numerous studies show that the cause of infection in people with various tick-borne infections are nymphs. This is due to their relatively small size, which makes them difficult to detect and, thus, they stay on the host longer before being removed. This also increases the chance of transmitting pathogens from the nymphs to the host [53,54,55]. The seasonal phenology of *I. ricinus* nymphs differs among geographic locations, but in continental Europe it is bimodal and consists of a large spring peak followed by a smaller autumn peak [56,57]. In this study, due to the relatively small number of collected nymphs, we could not demonstrate a pattern of nymphal activity. From a public health viewpoint, understanding the ecological factors that cause inter-annual variation in the NQI and, hence, in the risk of infection, it is important for developing control strategies to reduce the incidence of tick-borne disease.

Since only the NQI and TM showed statistically significant differences between urbanized and wild areas, we cannot claim that the risk for tick bite to humans is different in the two areas. Nymphs showed statistically higher levels in wild areas, but it is difficult to conclude the risk to humans due to their incredibly small sample size. On the other hand, the higher TM values obtained in the wild areas suggest that a contact with a tick will occur in a shorter time. Based on these data only, we cannot conclude that there is a difference in the distribution and density of tick populations between U and W. These differences are not sufficient to suggest that there is a significant difference in tick populations between both types of zones in Kaylaka Park. Further research involving more consistent sampling across space and time, multiple sampling methods and the adding of new sampling sites would provide a better estimate of tick populations in Kaylaka Park.

Practical methods to control ticks in large areas have not been developed. To reduce the incidence of tick-borne disease in endemic areas, public health efforts should, therefore, be focused on identifying areas that present a high risk for acquiring tick bites. In this study, we found the presence of hard ticks of the species *Ixodes ricinus* in all surveyed areas of Kaylaka Park, and their distribution did not differ significantly. These results indicate that regardless of the type of area surveyed, there is a high risk of tick bites and, consequently, of contracting tick-borne diseases. Kaylaka Park attracts many citizens, tourists and visitors and, therefore, effective measures need to be implemented to reduce the tick abundance in urban areas.

## Figures and Tables

**Table 1:** Geographical coordinates and a brief description of the land class.

Sampling Site	Latitude	Longitude	Altitude (m)	Size of the Sampling Site (m^2^)	Land Class
**U1**	43.3886736	24.6251901	154.90	730	Mainly on maintained lawn areas. However, the lawn is speckled with tree stands with leaf litter and understory vegetation, and some meadow areas, where flagging was conducted. Both deciduous and coniferous trees are present, with deciduous tree stands dominating. The area contains no forests.
**U2**	43.3856494	24.6257367	139.66	530	Parking for cars and lawns. Recreation and sports area with sycamore trees (*Platanus* spp.)
**U3**	43.3837788	24.6313879	127.38	440	The zoo, restaurant, cafes, playgrounds, open lawns with deciduous trees in the periphery. The zoo breeds deer (*Cervus elaphus*), fallow deer (*Dama dama*), a large population of African pigmy goat (*Capra aegagrus hircus*), mouflon (*Ovis gmelini*), brown bear (*Ursus arctos*) and various birds.
**U4**	43.379304	24.623335	152.35	520	Abandoned parachute platform, area for rock climbing, meadows, swimming pool.
**W1**	43.375829	24.625912	147.55	450	Nature-orientated part of the park, surrounded by high limestone cliffs in the karst valley of the Touchenitsa River. More forest-like structures with higher tree density, underbrush and wild animals such as roe deer exist in this part of Kaylaka Park. The lawns are mowed much less often and, therefore, areas with longer grass and bushes exist and park visitors have to stay on the walkways. Abandoned arboretum.
**W2**	43.345075	24.652153	192.88	520	Area around of the hut “Srebrostruy”, poorly maintained sports ground, a camping site and an adjoining car park. The area has dense mixed forest, high humidity and many animal species.
**W3**	43.383098	24.626121	179.98	460	The area around the ancient Roman fortress Strogozia, located above the river canyon. Narrow path between deciduous trees, open lawns are not maintained. Plenty of elderberry (*Sambucus nigra*) and ivy (*Hedera helix*). It has a weak presence of domestic animals such as strays dogs, and large mammals such as roe deer often pass.
**W4**	43.341813	24.6437243	253.20	4000	Protected forest territory “Bohotska Gora”, located southeast of the city of Pleven. Total area of 46.1 ha. The forest consists mainly of deciduous trees species such as oak (*Quercus* sp.), beech (*Fagus sylvatica*). The shrub layer includes mainly hawthorn (*Crataegus* sp.), smoke bush (*Cotinus* sp.) and rosehip (*Rosa canina*). Large wild mammals such as roe deer, wild boar, jackals and foxes exist.

**Table 3:** Collected ticks (*n*) in urbanized and wild sampling sites over the 5 years by 10 days period from March to June.

Period	Area Type	2016	2017	2018	2019	2020	Total
**1–10 March**	U	-	-	-	7	-	7
	W	-	-	-	4	-	4
**11–20 March**	U	-	-	13	2	-	15
	W	12	-	-	-	-	12
**21–31 March**	U	-	-	23	15	-	38
	W	-	-	-	-	-	0
**1–10 April**	U	25	-	-	1	-	26
	W	4	-	39	-	-	43
**11–20 April**	U	-	-	-	20	-	20
	W	-	-	-	-	-	0
**21–30 April**	U	-	-	11	12	-	23
	W	-	-	-	79	18	97
**1–10 May**	U	-	15	-	-	-	15
	W	61	35	56	46	-	198
**11–20 May**	U	-	-	-	-	-	0
	W	-	-	-	17	52	69
**21–30 May**	U	-	-	-	23	-	23
	W	-	-	-	-	-	0
**1–10 June**	U	18	-	-	3	-	21
	W	-	-	-	-	-	0
**11–20 June**	U	-	-	-	5	-	5
	W	-	-	-	-	-	0
**21–31 June**	U	-	-	-	6	-	6
	W	-	-	-	-	-	0
**Total**		120	50	142	240	70	622

U: urban areas; W: wild areas; -: no ticks collected.

**Table 4:** Statistically significant differences (*p* < 0.05) between urban and wild areas (Mann–Whitney U-test).

Areas	Number of Flagging Event (N)	TQI	AQI	NQI	TM
**U1–U3**	N_U1_ = 7 N_U3_ = 5	NS	NS	NS	*U* = 5.000 *Z* = −2.037 *p* = 0.042
**U1–W1**	N_U1_ = 7 N_W1_ = 3	NS	NS	*U* = 2.000 *Z* = −1.961 *p* = 0.05	*U* = 0.000 *Z* = −2.400 *p* = 0.016
**U1–W2**	N_U1_ = 7 N_W2_ = 6	*U* = 7.00 *Z* = −2.00 *p* = 0.046	*U* = 6.000 *Z* = 2.14 *p* = 0.032	NS	*U* = 5.000 *Z* = −2.289 *p* = 0.022
**U1–W4**	N_U1_ = 7 N_W4_ = 3	NS	NS	NS	*U* = 0.000 *Z* = −2.400 *p* = 0.016
**U2–W1**	N_U2_ = 9 N_W1_ = 3	NS	NS	NS	*U* = 0.000 *Z* = −2.501 *p* = 0.012
**U2–W2**	N_U2_ = 9 N_W2_ = 6	NS	*U* = 9.000 *Z* = −2.121 *p* = 0.034	NS	NS
**U2–W4**	N_U2_ = 9 N_W4_ = 3	NS	NS	NS	*U* = 1.500 *Z* = −2.227 *p* = 0.026
**U3–W1**	N_U3_ = 5 N_W1_ = 3	NS	NS	*U* = 0.000 *Z* = −2.382 *p* = 0.017	*U* = 0.000 *Z* = −2.249 *p* = 0.024
**U3–W2**	N_U3_ = 5 N_W2_ = 6	NS	NS	*U* = 3.000 *Z* = −2.298 *p* = 0.022	NS
**U3–W4**	N_U3_ = 5 N_W4_ = 3	NS	NS	NS	*U* = 0.000 *Z* = −2.249 *p* = 0.024
**W1–W3**	N_W1_ = 3 N_W3_ = 4	NS	NS	*U* = 0.000 *Z* = 2.141 *p* = 0.032	NS
**W2–W3**	N_W2_ = 6 N_W3_ = 4	*U* = 2.000 *Z* = −2.132 *p* = 0.033	*U* = 2.000 *Z* = −2.132 *p* = 0.033	NS	NS

U1–U4: urban areas1–4; W1–W4: wild areas1–4; TQI: total number of ticks/sampled area in m^2^; AQI: number of adult (male and female) ticks/sampled area in m^2^; NQI: number of nymph/sampled area in m^2^; TM: total number of ticks/minute; NS: not significant.

## Data Availability

The authors confirm that the data supporting the findings of this report are available within the article.
